# Therapeutic angiogenesis of adipose-derived stem cells for ischemic diseases

**DOI:** 10.1186/s13287-017-0578-2

**Published:** 2017-06-05

**Authors:** Lina Zhao, Takerra Johnson, Dong Liu

**Affiliations:** Cardiovascular Research Institute, Morehouse School of Medicine, 720 Westview Drive SW, Atlanta, GA 30310 USA

**Keywords:** Adipose-derived stem cells, Angiogenesis, Proangiogenic effect, Ischemic diseases

## Abstract

Ischemic diseases, the leading cause of disability and death, are caused by the stenosis or obstruction of arterioles/capillaries that is not compensated for by vessel dilatation or collateral circulation. Angiogenesis is a complex process leading to new blood vessel formation and is triggered by ischemic conditions. Adequate angiogenesis, as a compensatory mechanism in response to ischemia, may increase oxygen and nutrient supplies to tissues and protect their function. Therapeutic angiogenesis has been the most promising therapy for treating ischemic diseases. In recent years, stem cell transplantation has been recognized as a new technique with therapeutic angiogenic effects on ischemic diseases. Adipose-derived stem cells, characterized by their ease of acquisition, high yields, proliferative growth, and low immunogenicity, are an ideal cell source. In this review, the characterization of adipose-derived stem cells and the role of angiogenesis in ischemic attack are summarized. The angiogenic effects of adipose-derived stem cells are discussed from the perspectives of in-vitro, in-vivo, and clinical trial studies for the treatment of ischemic diseases, including ischemic cardiac, cerebral, and peripheral vascular diseases and wound healing. The microvesicles/exosomes released from adipose-derived stem cells are also presented as a novel therapeutic prospect for treating ischemic diseases.

## Background

Ischemia is a reduction in the blood supply, causing a shortage of oxygen and nutrients needed for cellular metabolism. The primary cause of ischemia is a blockage in the arterial blood supply to a tissue, organ, or extremity. Ischemic diseases are a group of diseases caused by ischemic injury, including ischemic heart disease, ischemic cerebral disease, and peripheral ischemic vascular disease, which are a worldwide public health issue and contribute to increases in mortality and disability.

Angiogenesis is the process of new blood vessels forming from pre-existing vessels and the subsequent expansion of the vascular network in the body. The body balances angiogenesis by producing precise amounts of proangiogenic factors, such as vascular endothelial growth factor (VEGF), basic fibroblast growth factor (bFGF), transforming growth factor beta (TGF-β), and platelet-derived endothelial cell growth factor, and anti-angiogenic factors, such as endostatin, angiostatin, tissue inhibitors of metalloproteinases, and IFN-α, under normal physiological conditions. This homeostatic relationship can be disturbed, and tissue hypoxia can stimulate angiogenesis, which works to increase the production of proangiogenic factors.

For decades, moving therapeutic angiogenesis using traditional cytokine-based and cell-based therapies from the bench to clinical trials has been a focus of medical research. Traditional cytokine-based therapies, including those that use VEGF, FGF, hepatocyte growth factor (HGF), and stromal cell-derived factor-1α, have shown theoretical and experimental promise for use in treating ischemic diseases [1]. Unfortunately, VEGF, the most promising proangiogenic factor, and other angiogenic factors have not been shown to be effective treatments in a variety of preclinical and clinical studies. In the placebo-controlled, double-blind VEGF in Ischemia for Vascular Angiogenesis (VIVA) trial [2], the administration of recombinant human VEGF via intracoronary and intravenous infusions to patients with stable exertional angina showed no difference when compared with the administration of a placebo for improvements in myocardial perfusion and primary end points. Cell-based therapy is a promising and developing new option for treating ischemic diseases. Stem cells are a class of undifferentiated cells with the ability to differentiate into specialized cell types and can be derived from bone marrow, peripheral blood, adipose tissue, cardiac tissue, muscle tissue, and other sources. Stem cell-based therapeutic approaches involve bone marrow-derived mononuclear cells and their subsets, such as mesenchymal stem cells and adipose-derived stem cells (ASCs), as well as endothelial progenitor cells, cardiac tissue-derived stem cells, and combinations of different cell types. Stem cell-based therapies have been shown to promote angiogenesis and to be effective in treating ischemic diseases [3]; however, there are still a great variety of challenges, including the variability and complexity of the cells, limitations on the amount of product that can be obtained, and product storage and distribution, among other factors. Studies researching the therapeutic effect of human hematopoietic stem cells in ischemic patients suggest that it requires at least 10^7^–10^9^ endothelial progenitor cells from peripheral blood for angiogenic cell therapy. Unfortunately, isolating this many of these rare endothelial progenitor cells requires a large amount of human blood, and thus they are not available for clinical application [4]. A study comparing stem cells derived from adipose tissue with those derived from peripheral blood revealed that a higher yield can be obtained from adipose tissue [5]. Furthermore, ASCs show a stronger proliferative potential than bone marrow-derived stem cells [6]. Additionally, with the increase of obesity in the population and the popularization of liposuction operations, adipose tissue is much easier to obtain than other tissues. The cell source is also a challenge in stem cell-based therapies, which includes autologous and allogeneic cell sources. In order to avoid immune rejection or alloreactivity and immunosuppression, an autologous cell source is better than an allogeneic one. However, limitations on the number of cells available and indetermination on the quality of autologous stem cells pose challenges to this undertaking. In studies examining the immunoregulatory properties of ASCs, researchers have concluded that culture-expanded ASCs do not induce allospecific T-cell proliferative responses because of their reduced expression of surface histocompatibility antigens. These studies suggest that ASCs have a distinct advantage in allotransplantation therapy compared with the use of other cell sources [7]. Thus, ASCs represent a good prospect for cell-based angiogenic therapies for clinical application.

## Characterization of ASCs

There have been several different nomenclatures for cell populations isolated from adipose tissue, such as “adipose stromal cells”, “adipose-derived adult stem cells”, and “adipose mesenchymal stem cells”. To address this confusion, the International Fat Applied Technology Society reached a consensus and adopted the term “adipose-derived stem cells” to identify the plastic-adherent, multipotent cell population isolated from adipose tissue [8]. Freshly isolated ASCs can be cultured on plastic dishes in the presence of fetal bovine serum with a cell doubling time of 2–4 days depending on the culture medium and passage number [9]. Like other stem cells, ASCs have the capability to differentiate into different cell lineages, such as endothelial cells (ECs), cardiomyocytes, neurons, smooth muscle cells, hepatocytes, adipocytes, osteocytes, and chondrocytes.

In addition to their multidifferentiation potential, ASCs can have therapeutic effects that rely on paracrine secretion. ASCs secrete several cytokines, growth factors, and chemokines that modulate angiogenesis and immune responses and facilitate the regeneration of damaged tissues. Rehman et al. [10] examined the production of paracrine factors by human ASCs and found that ASCs promote angiogenesis by producing VEGF, HGF, and TGF-β, and that VEGF secretion increases fivefold when ASCs are cultured under hypoxic conditions. Moreover, ASCs have been reported to improve blood flow in a mouse model of hindlimb ischemia by secreting growth factors, including VEGF and HGF, and they have antiapoptotic effects on cardiomyocytes via the production of VEGF and insulin-like growth factor-1 [11, 12].

## Pathological angiogenesis in ischemic diseases

Angiogenesis is commonly quiescent in a healthy adult and is generally only activated when hypoxic, inflammatory, or malignant conditions arise. Hypoxia upregulates the transcriptional activation of proangiogenic factors and affects ECs and vascular smooth muscle cells by reducing cell proliferation rates and triggering cell apoptosis. Therefore, angiogenesis—a necessary compensatory mechanism for responding to ischemia that is impaired and insufficient in ischemic diseases—must be restored and enhanced.

## Proangiogenic effects of ASCs for treating ischemic diseases

### Ischemic heart disease

Cardiovascular disease is a major health concern in the United States, and it causes almost one-third of all deaths globally. Ischemic heart disease, also known as coronary heart disease, is caused by the stenosis and occlusion of the coronary artery, which leads to insufficient blood supply to the heart and, subsequently, to myocardial hypoxia. Stem cell therapy, especially ASC-based therapy, has been a promising treatment strategy. Autologous ASCs were administered in a porcine model of myocardial infarction (MI) via intracoronary injection, and the results show that ASC implantation improves cardiac function and prevents adverse ventricular remodeling by increasing angiogenesis in the infarct border zone. Immunohistochemical analysis of the surface markers revealed that the ASCs differentiated into ECs and vascular smooth muscle cells after implantation [13]. Similar cardioprotective effects have been reported in other animal MI models, highlighting the multidifferentiation potential of ASCs, which has shown them capable of differentiating into ECs in a rat MI model [14] and into cardiomyocytes in a mouse MI model [15].

Mechanistic studies of the therapeutic effects of ASCs have shown that paracrine secretion is responsible for the therapeutic angiogenesis that occurs in the ASC-treated hearts. Wang et al. [16] assessed the therapeutic efficacy of ASCs on infarcted rat hearts, and their results show that ASCs improve cardiac function, prevent ventricular remodeling, and enhance angiogenesis in the infarct border zone after MI. However, only 0.5% of the engrafted ASCs were found to express cardiac-specific markers, and there has been no reported evidence of ASCs developing into cells that show contractile activity. The expression of growth factors, including VEGF, HGF, and insulin-like growth factor-1, in ASCs was upregulated under normal culture conditions in vitro, and the expression of VEGF was upregulated in the infarct border zone of ASC-treated hearts. The results indicate that the benefits observed in ASC-treated hearts can be attributed to paracrine effects of engrafted ASCs [16]. Moreover, paracrine effect was also found in heterografts of human ASCs in a rat MI model, which resulted in noticeable improvements in cardiac function and the prevention of myocardial remodeling via secretion of the proangiogenic factors VEGF, HGF, bFGF, nerve growth factor beta, and stromal cell-derived factor-1α [17].

## The proangiogenic effect of pretreated ASCs

Certain pretreatments for ASCs before transplantation also modify the cardioprotective and proangiogenic effects of ASCs. S-nitroso-*N*-acetyl-d,l-penicillamine (SNAP), a nitric oxide donor, can induce the differentiation of ASCs into cardiomyocytes in vitro. Berardi et al. [18] compared the therapeutic effects of SNAP-treated ASCs and untreated ASCs transplanted into a rat MI model, and found that SNAP-treated ASCs improved cardiac function and increased angiogenesis compared with the effects of untreated ASCs. Exendin-4, a native glucagon-like peptide 1 analog with insulinotropic properties, can protect ASCs against impaired adhesion and cell apoptosis. Pretreatment of ASCs with exendin-4 may promote the survival and engraftment of ASCs, and has been shown to induce cardiac differentiation in ASCs after transplantation in a rat MI model. Transplantation of exendin-4-pretreated ASCs in an MI model significantly improved left ventricular function while also reducing the infarct area and enhancing angiogenesis [19]. Melatonin pretreatment has been shown to promote the survival of transplanted ASCs and enhance their therapeutic potency in a rat MI model by stimulating paracrine secretion of proangiogenic factors from ASCs [20].

Three-dimensional (3D) cell culturing is a novel technique in stem cell research. 3D cell cultures better mimic in-vivo microenvironments and increase cell-to-cell interactions compared with 2D cultures [21]. Kim et al*.* studied the therapeutic potential of 3D-cultured human ASCs in a rat MI model and found that 3D spheroid culturing enhanced hASC paracrine secretion of VEGF, increasing VEGF protein levels fivefold compared with levels in normally cultured hASCs, and promoted differentiation into endothelial and smooth muscle cell lineages. The transplantation of 3D-cultured hASCs also reduced infarct size and cardiomyocyte apoptosis compared with the effects observed in the traditional 2D-cultured hASC-injected group [22].

Based on the evidence for the cardioprotective effects of ASCs from animal MI or ischemia/reperfusion models, the multidifferentiation capacity and the paracrine effects of ASCs make this cell type a promising candidate for treating ischemic heart disease (Table 1).

**Table 1:** Therapeutic angiogenic effect of ASCs in animal IHD models

Animal model	Cell source	Delivery	Outcome	Underlying mechanisms
Pig MI	Autologous ASCs	Intracoronary injection	LVEF↑, LV wall thickness↑, vascular density↑	Differentiation into ECs and SMCs
Rat MI	Rat ASCs	Intramyocardial injection	LVEF↑, LVFS↑, capillary vessels↑	Differentiation into ECs
Rat MI	Allogeneic SVF cells	Intramyocardial injection	LVEF↑, LVFS↑, ESD↓, ESV↓, capillary density↑	Differentiation into ECs Paracrine secretion: VEGF, HGF, MCP-1, TIMP1, TIMP4
Rabbit MI	Rabbit ASCs	Intramyocardial injection	LVEF↑, peak + dP/dt↑, scar size↓, capillary density↑	Differentiation into cardiomyocytes
Mouse MI	Mouse ASCs	Intramyocardial injection	LVEF↑, LVEDV↑, LVESV↑, capillary density↑	Differentiation into cardiomyocytes
Rat MI	Rat ASCs	Intramyocardial injection	LVEF↑, LV wall thickness↑, capillary density↑, Percentage of viable myocardium↑,	Paracrine secretion: VEGF
Nude rat MI	Human ASCs	Intramyocardial injection	LVEF↑, LVFS↑, EDV↓, ESV↓ LV wall thickness↑, infarct size↓, capillary density↑	Paracrine secretion: VEGF, HGF, β-NGF
Nude rat MI	Human ASCs	Intramyocardial injection	LVEF↑, LVFS↑, infarct size↓, capillary density↑	Paracrine secretion: VEGF, bFGF, SDF-1α
Pig I/R	Autologous ASCs	Intramyocardial injection	LVEF↑, ESD↓, ESV↓, EDV↓, capillary density↑	Regulation of MMP/TIMP system
Rat MI	3D-cultured human ASCs	Intramyocardial injection	Infarct size↓, cardiomyocyte apoptosis↓, Capillary density↑	Differentiated into ECs and VSMCs Paracrine secretion: VEGF
Rat MI	Rat SVF cells	laid onto the epicardium	Relative infarcted volume↓, coronary vascular density↑, perfused vessels↑	Paracrine secretion: VEGF
Pig I/R	Allogeneic ASCs	Intracoronary injection	Vascular density↑	Differentiation into VSMCs, ECs, and cardiomyocytes Paracrine secretion: VEGF

*↑* increase, *↓* decrease, *ASC* adipose-derived stem cell, *IHD* ischemic heart disease, *I/R* ischemia/reperfusion, *SVF* stromal vascular fraction, *SMC* smooth muscle cells, *LVFS* left ventricular fractional shortening, *ESD* end-systolic dimension, *ESV* end-systolic volume, *EDV* end-diastolic volume, *bFGF* basic fibroblast growth factor, *EC* endothelial cell, *EF* ejection fraction, *HGF* hepatocyte growth factor, *LV* left ventricular, *MI* myocardial infarction, *MMP* matrix metalloproteinase, *TIMP* tissue inhibitor of matrix metalloproteinase, *VEGF* vascular endothelial growth factor

## Ischemic cerebral disease

Ischemic stroke is caused by lack of blood supply to the brain, which occurs when cerebral blood vessels become narrowed or clogged with an atherosclerotic plaque or thromboembolism. During ischemic stroke, the reduction of oxygen supply in the ischemic area leads to compensatory angiogenesis to meet metabolic demands; thus, adequate angiogenesis is positively correlated with the survival rate of stroke patients [23]. Therefore, the promotion of angiogenesis in the ischemic area could be an important therapeutic target for treating ischemic stroke. The therapeutic efficacy of hASC-conditioned medium has been reported in a rat ischemic stroke model. The continuous infusion of conditioned medium into the lateral ventricle reduces infarction volumes and neural cell apoptosis, promotes EC proliferation, and increases microvessel density [24]. The intravenous administration of allogeneic ASCs in rats with permanent middle cerebral artery occlusion protects cerebral function, reduces brain cell death, and promotes angiogenesis and neurogenesis by increasing the secretion of VEGF, neurofilaments, and synaptophysin [25].

To compare the safety and efficacy of the administration of xenogeneic and allogeneic ASCs, hASCs and rat ASCs were intravenously injected into a rat ischemic stroke model. Compared with the controls, both xenogeneic ASCs and allogeneic ASCs produced no side effects, and they were equally effective with regard to functional recovery, the prevention of ischemic brain damage, and the enhancement of angiogenesis and synaptogenesis [26].

## Peripheral vascular disease

Peripheral vascular disease is a narrowing or blockage of the blood vessels other than those that supply blood to the heart or brain. Ischemic limb disease is the most common peripheral vascular disease and can result in permanent disability, amputation, and even death.

The administration of autologous ASCs in a mouse model of ischemic limb disease shows that the transplantation of ASCs results in a more rapid recovery of blood flow and increases in capillary density in ischemic muscle tissue [11]. The angiogenic potential of xenogeneic (human) ASCs was also assessed in a mouse model of hindlimb ischemia, and the transplantation of hASCs was shown to promote recovery from ischemic muscle injury and to increase capillary density via paracrine secretion of angiogenic molecules, such as growth-regulated oncogene, placental growth factor, experimental autoimmune neuritis-78, monocyte chemoattractant protein-1, interleukin-6, and interleukin-8 [27]. Fan et al. [28] analyzed the distribution and kinetics of engrafted ASCs after transplantation in a mouse hindlimb ischemia model using a 3D multimodality imaging technique and found that ASCs exert proangiogenic effects via a VEGF/mechanistic target of rapamycin/Akt-dependent pathway.

FGF-2 is reported to enhance hASC proliferation and reduce cell apoptosis under hypoxic conditions. The addition of FGF-2 to the culture medium of hASCs promotes paracrine secretion of angiogenic growth factors from hypoxic hASCs, including HGF, VEGF, endogenous FGF-2, and hypoxia-inducible factor-1α (HIF-1α). The local delivery of FGF-2 promotes the survival of hASCs transplanted into ischemic regions of mouse hindlimbs. Following hASC transplantation to an ischemic region, the local delivery of FGF-2 enhances mRNA expression levels of human HGF, VEGF, and FGF-2. Furthermore, a combined hASC and FGF-2 treatment shows greater improvements in blood perfusion, higher capillary densities, and enhanced inhibition of muscle degeneration and fibrosis in ischemic muscles than treatments with hASCs or FGF-2 alone [29]. Moreover, the proangiogenic effect of ASCs in a limb ischemia model can also be enhanced by different types of preconditioning, such a mitochondrial reactive oxygen species (ROS) generation [30], treatment with a low-level laser [31], and 3D spheroid culturing [32].

## Wound healing

Wound healing is the natural restoration of tissue following injury, and consists of a complicated, systematic process that includes four overlapping phases: hemostasis, inflammation, proliferation, and maturation. Angiogenesis, a vital process for wound healing, occurs during the proliferative phase and enables the supply of blood to sustain tissue restoration and enhance blood flow to carry healing factors to the wound.

The administration of ASCs to a mouse model of full-thickness wounds made using a dermal punch biopsy on the back via intramuscular injections around the wounded area has been shown to promote dermal wound healing, improve blood perfusion to the skin, and increase capillary density and VEGF plasma levels. These beneficial effects may rely on different mechanisms, including the differentiation of ASCs toward keratinocytes, paracrine secretion of angiogenic factors, and fusion with epidermal cells [33]. A study using ASCs transplanted in a diabetic rat model with full-thickness circular excisional wounds via intradermal injections demonstrated that ASCs accelerate wound healing rates and decrease the number of fibroblasts compared with the effects in the control group [34]. Additionally, there is abundant evidence for the therapeutic effects of ASCs on wound healing [35, 36] and specifically for diabetic wound healing [37, 38].

## Genetic modification promotes the proangiogenic effects ASCs

The therapeutic angiogenic effects of ASCs may be enhanced by pretreatments involving genetic modification. The genetic modification of ASCs is achieved by inducing specific gene expression patterns in a precise and well-controlled manner via various methodologies to fully exploit their potential. Song et al. introduced v-myc into hASCs using a lentiviral gene transfer system to generate v-myc-expressing hASCs (v-myc hASCs), and found that v-myc hASCs have stronger proliferation and migration potentials and secrete more VEGF than normal hASCs. They then examined the therapeutic effect of the culture medium from genetically modified hASCs for treating a cutaneous wound animal model and found that this culture medium accelerated wound healing by promoting angiogenesis [39]. Shevchenko et al*.* transduced hASCs using a recombinant adenoassociated virus encoding human VEGF165 and obtained modified hASCs with substantially increased levels of VEGF secretion (VEGF-hASCs). The transplantation of VEGF-hASCs in a murine ischemic hindlimb model increased angiogenesis and enhanced reperfusion and revascularization compared with the effects of normal hASCs [40]. Therefore, genetic modifications in cell-based angiogenic therapy may become a promising and novel strategy for treating ischemic diseases.

In addition to the aforementioned therapeutic effects, ASCs have also been reported to promote angiogenesis in treatments for ischemic colitis [41], ischemic colonic anastomoses [42], and ischemic kidney injury [43]. This evidence for the therapeutic angiogenic effects of ASCs suggests that this cell type is a promising candidate for use in the treatment of ischemic diseases.

## The application of ASCs in clinical trials

Based on the evidence for the therapeutic angiogenic effects of ASCs in animal ischemic disease models, the use of ASC treatments has moved on to clinical trials. Data on the ClinicalTrials.gov website show that there is a large number of clinical trials testing ASC-based treatments for ischemic diseases (Table 2). In this review, only completed and published trials are discussed.

**Table 2:** Clinical trials of ASCs in therapeutic angiogenesis for ischemic diseases

Patients with disease	Number of patients/follow-up	Phase	Type of cells	Cell quantity	Delivery route	ClinicalTrials.gov identifier
Ischemic cardiomyopathy	27 patients/36 months	I	Autologous ASCs	0.4 × 10^6^ cells/kg 0.8 × 10^6^ cells/kg 1.2 × 10^6^ cells/kg	Catheter-based transendocardial injection	NCT00426868
ST-elevation acute myocardial infarction	14 patients/6 months	I	Autologous ASCs	N/A	N/A	NCT00442806
Critical limb ischemia	13 patients/6 months	I/II	Autologous ASCs	100 × 10^6^ cells	Intramuscular injection	NCT01211028
Acute myocardial infarction	23 patients/36 months	II	ASCs	N/A	Intracoronary route	NCT01216995
Critical limb ischemia with diabetes	33 patients/12 months	I/II	Autologous ASCs	0.5 × 10^6^ cells/kg 1 × 10^6^ cells/kg	Intra-arterial administration through femoral artery	NCT01257776
Buerger’s disease	15 patients/24 weeks	I/II	Autologous ASCs	5 × 10^6^ cells/kg	Intramuscular infusion	NCT01302015
Lower limb ischemia	60 patients/12 months	I/II	Autologous ASCs	N/A	Coat the inside of a synthetic graft	NCT01305863
Chronic ischemic heart disease	60 patients/6 months	II	ASCs	N/A	N/A	NCT01449032
Chronic myocardial ischemia	28 patients/12 months	II	ASCs	0.4 × 10^6^ cells/kg (≤40 × 10^6^ cells)	Intramyocardial injection	NCT01556022
Critical limb ischemia	20 patients/48 weeks	I/II	Autologous ASCs	(100–300) × 10^6^ cells	Intramuscular injection	NCT01663376
Ischemic stroke	20 patients/24 months	II	Allogeneic ASCs	1 × 10^6^ cells/kg	Intravenous administration	NCT01678534
Ischemic heart failure	6 patients/6 months	N/A	Autologous ASCs	N/A	Intracoronary administration	NCT01709279
Critical limb ischemia	33 patients/12 months	I/II	Allogeneic ASCs	0.5 × 10^6^ cells/kg 1 × 10^6^ cells/kg	Administered intra-arterially	NCT01745744
Critical ischemia of the lower limbs	10 patients/1 year	I/II	Human ASCs	1 × 10^6^ cells/kg	Intramuscular injection	NCT01824069
Acute myocardial infarction	10 patients/6 months	I/II	Autologous ASCs	N/A	Implantation by a catheter delivery system and/or intravenously	NCT01974128
Chronic myocardial ischemia	3 patients/12 months	II	ASCs	0.8 × 10^6^ cells/kg (≤80 × 10^6^ cells)	Intramyocardial injection	NCT02052427
Diabetic with critical limb ischemia	48 patients/6 months	I/II	Autologous ASCs	0.5 × 10^6^ cells	Intra-arterial infusion	NCT02287974
Ischemic heart disease and heart failure	10 patients/6 months	I	Allogeneic ASCs	100 × 10^6^ cells	Intramyocardial injection	NCT02387723
Deep second-degree burn wound	5 patients/4 weeks	I	ALLO-ASC-DFU	N/A	External application	NCT02394873
Diabetic foot ulcer	5 patients/4 weeks	I	ALLO-ASC-DFU, a hydrogel sheet containing allogeneic ASCs	N/A	External application	NCT02394886
Heart failure	138 patients/6 months	II	Allogeneic ASCs	100 × 10^6^ cells	Intramyocardial injection	NCT02673164
Peripheral arterial disease	10 patients/6 months	I/II	Autologous ASCs	200 × 10^6^ cells	Intravenous injection; intra-adventitia (proximal to the lesion) injection; intramuscular injection	NCT02756884
Chronic nonhemorrhagic stroke	3 patients/6 months	I	Autologous ASCs	N/A	Transplantation into brain surrounding ischemic infarct	NCT02813512
Diabetic foot	240 patients/3 months	I	ASCs	N/A	N/A	NCT02831075
Stroke	400 patients/6 months	II/III	Allogeneic ASCs	1 × 10^6^ cells/kg	Intravenous infusion	NCT02849613
Critical lower limb ischemia	9 patients/24 weeks	I/II	ASCs	N/A	Intramuscular injection	NCT02864654
Diabetic foot ulcer	45 patients/20 weeks	II	Autologous ASCs	N/A	Local treatment	NCT02866565

*ASC* adipose-derived stem cell, *N/A* not available

In a pilot study, 15 male patients with critical limb ischemia were treated with multiple intramuscular hASC injections; 11 patients were responsive to the hASC treatment. The pain rating scores after 6 months of hASC transplantation were significantly improved in 11 patients compared with the baseline scores. An enhancement of collateral vessel formation, examined via digital subtraction angiography, was observed in 10 patients [44]. A similar trial observed beneficial improvement without any complications [45]. These studies suggest that the transplantation of ASCs might be a safe, alternative approach to achieving therapeutic angiogenesis in critical limb ischemia patients.

A phase I prospective, randomized, placebo-controlled and double-blind trial assessed the safety and feasibility of the transplantation of autologous ASCs via transendocardial injections in patients with ischemic cardiomyopathy. Twenty-seven patients were enrolled in the study, including 21 ASC-treated patients and six control patients. There were no significant differences in cardiac-related deaths between the ASC-treated group and the control group, and no malignant arrhythmias were observed at the 36-month follow-up in either group. ASC treatment not only improved global wall motion score index values and prevented increases in left ventricular infarcted mass at 6 months compared with the effects in the control group, but also preserved the patients’ exercise tolerance and maximal oxygen consumption [46]. Recently, two phase II clinical trials (ATHENA I/II) testing the administration of ASCs on patients suffering from refractory chronic myocardial ischemia with left ventricular dysfunction have been reported [47]. The results demonstrated that intramyocardial delivery of ASCs reduced heart failure hospitalizations and improved angina symptoms, but did not improve left ventricular function or volumes at 12 months. These trials suggest that administration of ASCs via transendocardial injections is safe, feasible, and scalable in patients with ischemic cardiomyopathy.

Although most clinical trials are still in progress, the results are promising, and the outcomes of these trials may provide more evidence and experiences of ASC-based treatments for ischemic diseases.

## Microvesicles/exosomes from ASCs: from mediator to medicine

Although there is strong evidence demonstrating the therapeutic effect of ASCs, the mechanisms underlying these effects are still unclear. Present mechanistic studies have revealed two major mechanisms: multidifferentiation and paracrine secretion. A significant proportion of the benefits of ASC administration may be due to secretion rather than differentiation [11, 16]. The administration of conditioned medium from ASCs shows efficient therapeutic effects on ischemic diseases [48], which indicates that ASCs exert their function via secretion. Besides the secretion of growth factors and cytokines already discussed, microvesicles/exosomes are also reported to be released from ASCs.

Extracellular vesicles are a group of vesicles released by cells to the extracellular environment and may be classified as exosomes, microvesicles, apoptotic bodies, or other subsets according to their cellular origin and size. Extracellular vesicles have emerged as novel mediators of intercellular communication, being involved in the transmission of biological signals between cells to regulate various biological processes. Microvesicles/exosomes are plasma membrane-derived vesicles released by healthy cells and have been recognized as transmitters loaded with RNAs, proteins, and lipids that participate in intercellular communication. Microvesicles/exosomes can be taken up by neighboring or distant cells and can trigger downstream signaling events in recipient cells. The pathophysiological roles for microvesicles/exosomes were originally found in mechanistic studies of cells [49]. However, an emerging approach using microvesicles/exosomes for therapeutic angiogenesis may present a promising outlook for treating ischemic diseases [50]. Recent studies provide evidence that microvesicles/exosomes from ASCs may contribute to the therapeutic efficacy of ASCs [51]. Our laboratory has demonstrated the proangiogenic potential of ASC-released microvesicles and has shown that microRNA-31 in microvesicles contributes to these proangiogenic effects by targeting factor inhibiting HIF-1 (FIH-1) in recipient ECs [52]. Further studies in animal models with ischemic diseases are required to provide more evidence for the therapeutic efficacy of ASC-released microvesicles/exosomes. Currently, the only clinical trial using microvesicle/exosomes is a study of the effects of microvesicles/exosomes from umbilical cord blood-derived mesenchymal stem cells on β-cell mass and glycemic control in type 1 diabetes mellitus patients (ClinicalTrials.gov: NCT02138331). More clinical trials using microvesicles/exosomes need to be carried out.

Microvesicles/exosomes, as natural cell-derived extracellular vesicles, have recently been highlighted for use in a novel therapeutic strategy for the delivery of nucleotides, proteins, and chemical drugs in several diseases [53–55]. This strategy involves using engineered microvesicles/exosomes loaded with specific genes, proteins, or chemical drugs in a controlled manner, and delivering them into target cells to exert therapeutic effects. However, studies on using engineered microvesicles/exosomes from ASCs are rare, and there is a lack of evidence for the therapeutic efficiency of engineered microvesicles/exosomes in ischemic diseases.

## Conclusions

ASCs have emerged as a novel option for cell-based therapy because of their high yield and low immunogenicity. Based on strong evidence from basic research and clinical trials, ASCs have been reported as a promising cell type for use in the treatment of ischemic diseases, such as ischemic heart disease, ischemic cerebral disease, peripheral vascular diseases, and wound healing. The pretreatment of ASCs with biochemical or physical factors may enhance their proangiogenic effect. Meanwhile, different administration routes for ASCs may lead to differences in their therapeutic effect. Mechanistic studies have demonstrated that the therapeutic proangiogenic effects of ASCs are attributed more to their secretory potential than to their capacity for multidifferentiation. In addition to growth factors and cytokines, microvesicles/exosomes are also secreted by ASCs, presenting a novel approach for therapeutic angiogenesis in treating ischemic diseases. The evidence for microvesicle/exosome-based therapy is still insufficient, and further mechanistic studies and clinical trials are required. In addition, engineering microvesicles/exosomes may broaden their prospects for use in therapeutic strategies for treating ischemic diseases.

## Abbreviations

3D: Three-dimensional
ASC: Adipose-derived stem cell
EC: Endothelial cell
FGF: Fibroblast growth factor
HGF: Hepatocyte growth factor
HIF-1: Hypoxia-inducible factor 1
MI: Myocardial infarction
SNAP: S-nitroso-*N*-acetyl-d,l-penicillamine
TGF-β: Transforming growth factor beta
VEGF: Vascular endothelial growth factor

## References

[1] Atluri P, Woo YJ (2008). Pro-angiogenic cytokines as cardiovascular therapeutics: assessing the potential. BioDrugs.

[2] Henry TD, Annex BH, McKendall GR, Azrin MA, Lopez JJ, Giordano FJ, Shah PK, Willerson JT, Benza RL, Berman DS (2003). The VIVA trial: Vascular endothelial growth factor in Ischemia for Vascular Angiogenesis. Circulation.

[3] Borlongan CV (2016). Age of PISCES: stem-cell clinical trials in stroke. Lancet.

[4] Madonna R, Geng YJ, De Caterina R (2009). Adipose tissue-derived stem cells: characterization and potential for cardiovascular repair. Arterioscler Thromb Vasc Biol.

[5] Madonna R, De Caterina R (2008). *In vitro* neovasculogenic potential of resident adipose tissue precursors. Am J Physiol Cell Physiol.

[6] De Ugarte DA, Morizono K, Elbarbary A, Alfonso Z, Zuk PA, Zhu M, Dragoo JL, Ashjian P, Thomas B, Benhaim P (2003). Comparison of multi-lineage cells from human adipose tissue and bone marrow. Cells Tissues Organs.

[7] McIntosh K, Zvonic S, Garrett S, Mitchell JB, Floyd ZE, Hammill L, Kloster A, Di Halvorsen Y, Ting JP, Storms RW (2006). The immunogenicity of human adipose-derived cells: temporal changes *in vitro*. Stem Cells.

[8] Gimble JM, Katz AJ, Bunnell BA (2007). Adipose-derived stem cells for regenerative medicine. Circ Res.

[9] Mitchell JB, McIntosh K, Zvonic S, Garrett S, Floyd ZE, Kloster A, Di Halvorsen Y, Storms RW, Goh B, Kilroy G (2006). Immunophenotype of human adipose-derived cells: temporal changes in stromal-associated and stem cell-associated markers. Stem Cells.

[10] Rehman J, Traktuev D, Li J, Merfeld-Clauss S, Temm-Grove CJ, Bovenkerk JE, Pell CL, Johnstone BH, Considine RV, March KL (2004). Secretion of angiogenic and antiapoptotic factors by human adipose stromal cells. Circulation.

[11] Nakagami H, Maeda K, Morishita R, Iguchi S, Nishikawa T, Takami Y, Kikuchi Y, Saito Y, Tamai K, Ogihara T (2005). Novel autologous cell therapy in ischemic limb disease through growth factor secretion by cultured adipose tissue-derived stromal cells. Arterioscler Thromb Vasc Biol.

[12] Chen G, Xu C, Cen M (2016). TIEG1 suppression enhances the therapeutic efficacy of human adipose-derived mesenchymal stem cells in myocardial infarct repair. Heart Vessels.

[13] Valina C, Pinkernell K, Song YH, Bai X, Sadat S, Campeau RJ, Le Jemtel TH, Alt E (2007). Intracoronary administration of autologous adipose tissue-derived stem cells improves left ventricular function, perfusion, and remodelling after acute myocardial infarction. Eur Heart J.

[14] Mazo M, Cemborain A, Gavira JJ, Abizanda G, Arana M, Casado M, Soriano M, Hernandez S, Moreno C, Ecay M (2012). Adipose stromal vascular fraction improves cardiac function in chronic myocardial infarction through differentiation and paracrine activity. Cell Transplant.

[15] Leobon B, Roncalli J, Joffre C, Mazo M, Boisson M, Barreau C, Calise D, Arnaud E, Andre M, Puceat M (2009). Adipose-derived cardiomyogenic cells: *in vitro* expansion and functional improvement in a mouse model of myocardial infarction. Cardiovasc Res.

[16] Wang L, Deng J, Tian W, Xiang B, Yang T, Li G, Wang J, Gruwel M, Kashour T, Rendell J (2009). Adipose-derived stem cells are an effective cell candidate for treatment of heart failure: an MR imaging study of rat hearts. Am J Physiol Heart Circ Physiol.

[17] Cai L, Johnstone BH, Cook TG, Tan J, Fishbein MC, Chen PS, March KL (2009). IFATS collection: Human adipose tissue-derived stem cells induce angiogenesis and nerve sprouting following myocardial infarction, in conjunction with potent preservation of cardiac function. Stem Cells.

[18] Berardi GR, Rebelatto CK, Tavares HF, Ingberman M, Shigunov P, Barchiki F, Aguiar AM, Miyague NI, Francisco JC, Correa A (2011). Transplantation of SNAP-treated adipose tissue-derived stem cells improves cardiac function and induces neovascularization after myocardium infarct in rats. Exp Mol Pathol.

[19] Liu J, Wang H, Wang Y, Yin Y, Wang L, Liu Z, Yang J, Chen Y, Wang C (2014). Exendin-4 pretreated adipose derived stem cells are resistant to oxidative stress and improve cardiac performance via enhanced adhesion in the infarcted heart. PLoS ONE.

[20] Zhu P, Liu J, Shi J, Zhou Q, Liu J, Zhang X, Du Z, Liu Q, Guo Y (2015). Melatonin protects ADSCs from ROS and enhances their therapeutic potency in a rat model of myocardial infarction. J Cell Mol Med.

[21] Haycock JW (2011). 3D cell culture: a review of current approaches and techniques. Methods Mol Biol.

[22] Kim JH, Park IS, Park Y, Jung Y, Kim SH, Kim SH (2013). Therapeutic angiogenesis of three-dimensionally cultured adipose-derived stem cells in rat infarcted hearts. Cytotherapy.

[23] Wei L, Keogh CL, Whitaker VR, Theus MH, Yu SP (2005). Angiogenesis and stem cell transplantation as potential treatments of cerebral ischemic stroke. Pathophysiology.

[24] Cho YJ, Song HS, Bhang S, Lee S, Kang BG, Lee JC, An J, Cha CI, Nam DH, Kim BS (2012). Therapeutic effects of human adipose stem cell-conditioned medium on stroke. J Neurosci Res.

[25] Gutierrez-Fernandez M, Rodriguez-Frutos B, Ramos-Cejudo J, Teresa Vallejo-Cremades M, Fuentes B, Cerdan S, Diez-Tejedor E (2013). Effects of intravenous administration of allogenic bone marrow- and adipose tissue-derived mesenchymal stem cells on functional recovery and brain repair markers in experimental ischemic stroke. Stem Cell Res Ther.

[26] Gutierrez-Fernandez M, Rodriguez-Frutos B, Ramos-Cejudo J, Otero-Ortega L, Fuentes B, Vallejo-Cremades MT, Sanz-Cuesta BE, Diez-Tejedor E (2015). Comparison between xenogeneic and allogeneic adipose mesenchymal stem cells in the treatment of acute cerebral infarct: proof of concept in rats. J Transl Med.

[27] Moon MH, Kim SY, Kim YJ, Kim SJ, Lee JB, Bae YC, Sung SM, Jung JS (2006). Human adipose tissue-derived mesenchymal stem cells improve postnatal neovascularization in a mouse model of hindlimb ischemia. Cell Physiol Biochem.

[28] Fan W, Sun D, Liu J, Liang D, Wang Y, Narsinh KH, Li Y, Qin X, Liang J, Tian J (2012). Adipose stromal cells amplify angiogenic signaling via the VEGF/mTOR/Akt pathway in a murine hindlimb ischemia model: a 3D multimodality imaging study. PLoS ONE.

[29] Bhang SH, Cho SW, Lim JM, Kang JM, Lee TJ, Yang HS, Song YS, Park MH, Kim HS, Yoo KJ (2009). Locally delivered growth factor enhances the angiogenic efficacy of adipose-derived stromal cells transplanted to ischemic limbs. Stem Cells.

[30] Carriere A, Ebrahimian TG, Dehez S, Auge N, Joffre C, Andre M, Arnal S, Duriez M, Barreau C, Arnaud E (2009). Preconditioning by mitochondrial reactive oxygen species improves the proangiogenic potential of adipose-derived cells-based therapy. Arterioscler Thromb Vasc Biol.

[31] Park IS, Chung PS, Ahn JC (2014). Enhanced angiogenic effect of adipose-derived stromal cell spheroid with low-level light therapy in hind limb ischemia mice. Biomaterials.

[32] Lee JH, Han YS, Lee SH (2016). Long-duration three-dimensional spheroid culture promotes angiogenic activities of adipose-derived mesenchymal stem cells. Biomol Ther (Seoul).

[33] Ebrahimian TG, Pouzoulet F, Squiban C, Buard V, Andre M, Cousin B, Gourmelon P, Benderitter M, Casteilla L, Tamarat R (2009). Cell therapy based on adipose tissue-derived stromal cells promotes physiological and pathological wound healing. Arterioscler Thromb Vasc Biol.

[34] Maharlooei MK, Bagheri M, Solhjou Z, Jahromi BM, Akrami M, Rohani L, Monabati A, Noorafshan A, Omrani GR (2011). Adipose tissue derived mesenchymal stem cell (AD-MSC) promotes skin wound healing in diabetic rats. Diabetes Res Clin Pract.

[35] Zamora DO, Natesan S, Becerra S, Wrice N, Chung E, Suggs LJ, Christy RJ (2013). Enhanced wound vascularization using a dsASCs seeded FPEG scaffold. Angiogenesis.

[36] Bliley JM, Argenta A, Satish L, McLaughlin MM, Dees A, Tompkins-Rhoades C, Marra KG, Rubin JP (2016). Administration of adipose-derived stem cells enhances vascularity, induces collagen deposition, and dermal adipogenesis in burn wounds. Burns.

[37] Nie C, Zhang G, Yang D, Liu T, Liu D, Xu J, Zhang J (2015). Targeted delivery of adipose-derived stem cells via acellular dermal matrix enhances wound repair in diabetic rats. J Tissue Eng Regen Med.

[38] Zografou A, Papadopoulos O, Tsigris C, Kavantzas N, Michalopoulos E, Chatzistamatiou T, Papassavas A, Stavropoulou-Gioka C, Dontas I, Perrea D (2013). Autologous transplantation of adipose-derived stem cells enhances skin graft survival and wound healing in diabetic rats. Ann Plast Surg.

[39] Song SH, Lee MO, Lee JS, Jeong HC, Kim HG, Kim WS, Hur M, Cha HJ (2012). Genetic modification of human adipose-derived stem cells for promoting wound healing. J Dermatol Sci.

[40] Shevchenko EK, Makarevich PI, Tsokolaeva ZI, Boldyreva MA, Sysoeva VY, Tkachuk VA, Parfyonova YV (2013). Transplantation of modified human adipose derived stromal cells expressing VEGF165 results in more efficient angiogenic response in ischemic skeletal muscle. J Transl Med.

[41] Joo HH, Jo HJ, Jung TD, Ahn MS, Bae KB, Hong KH, Kim J, Kim JT, Kim SH, Yang YI (2012). Adipose-derived stem cells on the healing of ischemic colitis: a therapeutic effect by angiogenesis. Int J Colorectal Dis.

[42] Yoo JH, Shin JH, An MS, Ha TK, Kim KH, Bae KB, Kim TH, Choi CS, Hong KH, Kim J (2012). Adipose-tissue-derived stem cells enhance the healing of ischemic colonic anastomoses: an experimental study in rats. J Korean Soc Coloproctol.

[43] Xu Y, Shi T, Xu A, Zhang L (2016). 3D spheroid culture enhances survival and therapeutic capacities of MSCs injected into ischemic kidney. J Cell Mol Med.

[44] Lee HC, An SG, Lee HW, Park JS, Cha KS, Hong TJ, Park JH, Lee SY, Kim SP, Kim YD (2012). Safety and effect of adipose tissue-derived stem cell implantation in patients with critical limb ischemia: a pilot study. Circ J.

[45] Bura A, Planat-Benard V, Bourin P, Silvestre JS, Gross F, Grolleau JL, Saint-Lebese B, Peyrafitte JA, Fleury S, Gadelorge M (2014). Phase I trial: the use of autologous cultured adipose-derived stroma/stem cells to treat patients with non-revascularizable critical limb ischemia. Cytotherapy.

[46] Perin EC, Sanz-Ruiz R, Sanchez PL, Lasso J, Perez-Cano R, Alonso-Farto JC, Perez-David E, Fernandez-Santos ME, Serruys PW, Duckers HJ (2014). Adipose-derived regenerative cells in patients with ischemic cardiomyopathy: The PRECISE Trial. Am Heart J.

[47] Henry TD, Pepine CJ, Lambert CR, Traverse JH, Schatz R, Costa M, Povsic TJ, David Anderson R, Willerson JT, Kesten S (2017). The Athena trials: Autologous adipose-derived regenerative cells for refractory chronic myocardial ischemia with left ventricular dysfunction. Catheter Cardiovasc Interv.

[48] Bhang SH, Lee S, Shin JY, Lee TJ, Jang HK, Kim BS (2014). Efficacious and clinically relevant conditioned medium of human adipose-derived stem cells for therapeutic angiogenesis. Mol Ther.

[49] Dignat-George F, Boulanger CM (2011). The many faces of endothelial microparticles. Arterioscler Thromb Vasc Biol.

[50] Martinez MC, Andriantsitohaina R (2011). Microparticles in angiogenesis: therapeutic potential. Circ Res.

[51] Maredziak M, Marycz K, Lewandowski D, Siudzinska A, Smieszek A (2015). Static magnetic field enhances synthesis and secretion of membrane-derived microvesicles (MVs) rich in VEGF and BMP-2 in equine adipose-derived stromal cells (EqASCs)—a new approach in veterinary regenerative medicine. In Vitro Cell Dev Biol Anim.

[52] Kang T, Jones TM, Naddell C, Bacanamwo M, Calvert JW, Thompson WE, Bond VC, Chen YE, Liu D (2016). Adipose-derived stem cells induce angiogenesis via microvesicle transport of miRNA-31. Stem Cells Transl Med.

[53] Ohno S, Takanashi M, Sudo K, Ueda S, Ishikawa A, Matsuyama N, Fujita K, Mizutani T, Ohgi T, Ochiya T (2013). Systemically injected exosomes targeted to EGFR deliver antitumor microRNA to breast cancer cells. Mol Ther.

[54] Haney MJ, Klyachko NL, Zhao Y, Gupta R, Plotnikova EG, He Z, Patel T, Piroyan A, Sokolsky M, Kabanov AV (2015). Exosomes as drug delivery vehicles for Parkinson’s disease therapy. J Control Release.

[55] Yim N, Ryu SW, Choi K, Lee KR, Lee S, Choi H, Kim J, Shaker MR, Sun W, Park JH (2016). Exosome engineering for efficient intracellular delivery of soluble proteins using optically reversible protein-protein interaction module. Nat Commun.

